# Our Children

**Published:** 1901-12-07

**Authors:** 


### The Hospital

A Journal of

The Medical Sciences and Hospital Administration.

Vol. XXXI.—No. 793.] Edited by Sir Henry Burdett, K.C.B., and Solomon C. Smith, M.D., M.R.C.P. [December 7, 1901.

### Our Children.

A letter recently written by Earl Grey to the *Times*, in which he advocated the periodical weighing and measuring of children as a means of directing public attention to any decadence from whatever might be considered in each locality an adequate standard of height and weight at any given age, is one which seems to open out a somewhat more extended prospect than that which, to all appearance, was present before the mind of the writer. Lord Grey has convinced himself—we do not know upon what evidence—that the general development of town-bred children is inferior to that of their rustic contemporaries, or even that the inferiority is sufficient in degree seriously to threaten the physical future of a race which seems impelled by circumstances to resort to towns in a continually increasing proportion. His idea seems to be that a systematic half-yearly weighing and measuring in schools would not only serve to bring the differences between town and country children into prominence, but would also call public attention to these differences with sufficient force to compel the adoption of, or at least the search for, any remedies calculated to diminish conditions which he regards as threatening to the future of the Empire, and he appears to rest his hopes in this direction upon the fact that the careful weighing and measuring of certain domestic animals has enabled breeders to improve them in the respects in which they were shown to be defective. There can probably be no doubt that the systematic study of child development in every direction is a matter eminently worthy of all the pains and care which can be bestowed upon it, and our chief criticism of Lord Grey's proposal would be that it does not go far enough to obtain all the knowledge with regard to the young which would be accessible to inquiry, and which might be turned to good account if it were acquired. There seems to be something like a promise on the part of the Government, and there is certainly every expectation on the part of the public, of coming legislation with regard to schools which will be so framed as to profit by the errors or by the experience of the past 30 years, and to provide educational authorities which will not be merely old School Boards writ large, or collections of faddists bent upon carrying out their hobbies at the public expense, but will be composed, to whatever extent may be possible, of persons willing to be guided by any knowledge which can be placed at their disposal, or to assist in procuring any which may be conducive to the better fulfilment of their duties. If this expectation should be fulfilled, either in the next or in any near session of Parliament, it will, we think, have to be conceded that among the kinds of knowledge necessary for the proper guidance of education a prominent place must be given to physiology; and, if this be so, there will be an evident fitness in the suggestion that the new educational authorities should be largely recruited from the ranks of the medical profession.

It is not too much to say that a very large number of conditions common in childhood, and for which the stupid can think of no remedy but some form of punishment, have been shown to be nerve storms incidental to over-exertion, or to improperly directed exertion, of various centres, or to peripheral sources of irritation which might be removed by a little care and thoughtfulness, but which, when suffered to remain in operation, become sources of reflex phenomena which are described, in popular phraseology, under the one word "naughtiness." The head master of the great elementary school which formed the first link in the educational chain established some 30 years ago at Edinburgh, by the application to this purpose of the endowments of the old so-called "hospitals" of that city, was accustomed to declare that the secret of his exceptional success was that he "would have no naughty children and no boobies." In other words, the occasions of what would elsewhere have been called "naughtiness" were systematically withheld; and any child less quick at learning than its contemporaries was gently lifted to their level by kindly and considerate help from the teacher, and was never suffered to become an object of derision in the class. There are, we believe, at least two societies existing in England which have been established for the express purpose of the study of childhood, and in order to afford much-needed guidance to those who, either as parents or as instructors, are responsible for the care of the young, and Lord Grey could render no better service to his country than by endeavouring to nationalise such work as this by raising the societies in question to a more public position, and by assisting them to spread abroad the results of their labours, while the members of the medical profession might fitly co-operate in the task of applying to the schools and families of their respective localities a better knowledge of the essential requirements of childhood than is now generally possessed.

